# Development of an efficient cytosolic isobutanol production pathway in *Saccharomyces cerevisiae* by optimizing copy numbers and expression of the pathway genes based on the toxic effect of α-acetolactate

**DOI:** 10.1038/s41598-019-40631-5

**Published:** 2019-03-08

**Authors:** Seong-Hee Park, Ji-Sook Hahn

**Affiliations:** School of Chemical and Biological Engineering, Institute of Chemical Processes, Seoul National University, 1 Gwanak-ro, Gwanak-gu, Seoul 08826 Republic of Korea

**Keywords:** Metabolic engineering, Metabolic engineering

## Abstract

Isobutanol production in *Saccharomyces cerevisiae* is limited by subcellular compartmentalization of the pathway enzymes. In this study, we improved isobutanol production in *S*. *cerevisiae* by constructing an artificial cytosolic isobutanol biosynthetic pathway consisting of AlsS, α-acetolactate synthase from *Bacillus subtilis*, and two endogenous mitochondrial enzymes, ketol-acid reductoisomerase (Ilv5) and dihydroxy-acid dehydratase (Ilv3), targeted to the cytosol. *B*. *subtilis* AlsS was more active than Ilv2ΔN54, an endogenous α-acetolactate synthase targeted to the cytosol. However, overexpression of *alsS* led to a growth inhibition, which was alleviated by overexpressing *ILV5ΔN48* and *ILV3ΔN19*, encoding the downstream enzymes targeted to the cytosol. Therefore, accumulation of the intermediate α-acetolactate might be toxic to the cells. Based on these findings, we improved isobutanol production by expressing *alsS* under the control of a copper-inducible *CUP1* promoter, and by increasing translational efficiency of the *ILV5ΔN48* and *ILV3ΔN19* genes by adding Kozak sequence. Furthermore, strains with multi-copy integration of *alsS* into the delta-sequences were screened based on growth inhibition upon copper-dependent induction of *alsS*. Next, the *ILV5ΔN48* and *ILV3ΔN19* genes were integrated into the rDNA sites of the *alsS*-integrated strain, and the strains with multi-copy integration were screened based on the growth recovery. After optimizing the induction conditions of *alsS*, the final engineered strain JHY43D24 produced 263.2 mg/L isobutanol, exhibiting about 3.3-fold increase in production compared to a control strain constitutively expressing *ILV2ΔN54*, *ILV5ΔN48*, and *ILV3ΔN19* on plasmids.

## Introduction

Isobutanol is a promising biofuel candidate because of its higher energy density and lower hygroscopicity than ethanol^1,2^. Many bacterial species such as *Escherichia coli*, *Corynebacterium glutamicum*, and *Bacillus subtilis* have been engineered to produce isobutanol by introducing Ehrlich pathway enzymes involved in fusel alcohol formation from 2-keto acids^3^. In these bacteria, pyruvate is converted to α-acetolactate by α-acetolactate synthase (ALS) and then converted to 2-ketoisovalerate (2-KIV) by sequential catalytic reactions of ketol-acid reductoisomerase (KARI) and dihydroxy-acid dehydratase (DHAD). Finally, isobutanol is produced from 2-KIV via heterologous Ehrlich pathway enzymes, 2-ketoacid decarboxylase (KDC) and alcohol dehydrogenase (ADH)^4,5^.

**Figure 1:**
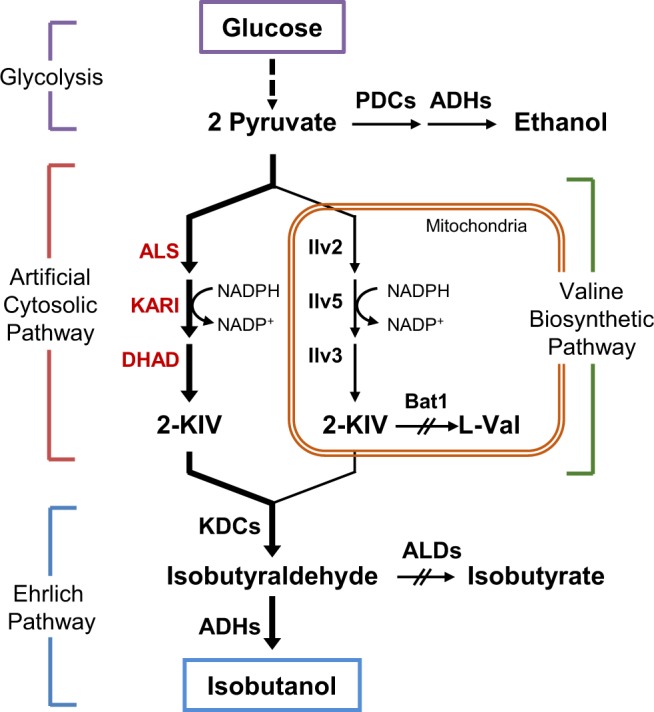
Schematic illustration of an artificial cytosolic isobutanol biosynthetic pathway. Pyruvate is converted to 2-ketoisovalerate (2-KIV) by sequential catalytic reactions of α-acetolactate synthase (ALS), ketol-acid reductoisomerase (KARI), and dihydroxy-acid dehydratase (DHAD), and then 2-KIV is converted to isobutanol by 2-ketoacid decarboxylase (KDCs) and alcohol dehydrogenase (ADH). Deleted gene is indicated by double slash (//) and dashed line indicate multiple enzymatic actions.

*Saccharomyces cerevisiae*, which can naturally produce isobutanol via valine catabolism (Fig. 1), is a promising host for the production of various alcohols due to its high alcohol tolerance^6,7^. There have been several efforts to increase isobutanol production in *S*. *cerevisiae*, but the production levels were still lower than those produced in the engineered bacteria^8–13^. Unlike bacteria, the isobutanol production pathway enzymes in *S*. *cerevisiae* are partitioned between the cytosol and the mitochondria (Fig. 1). In *S*. *cerevisiae*, cytosolic pyruvate is imported into the mitochondria via mitochondrial pyruvate carrier (MPC) complex^14,15^ and then pyruvate is converted to 2-KIV by sequential catalytic reactions of Ilv2 (ALS), Ilv5 (KARI), and Ilv3 (DHAD) in the mitochondrial matrix. The mitochondrial 2-KIV is then exported to the cytosol, and finally converted to isobutanol through Ehrlich pathway, involving endogenous KDCs and ADHs^4^. Therefore, subcellular compartmentalization of valine biosynthetic enzymes is one of the limiting factors for efficient isobutanol production in *S*. *cerevisiae*. To overcome this limitation, the whole isobutanol biosynthetic pathway has been relocated either to the cytosol by expressing the mitochondrial enzymes (Ilv2, Ilv3, and Ilv5) in the cytosol^8,13^ or to the mitochondria by expressing the cytosolic enzymes (KDCs and ADHs) in the mitochondria^16^. Previously, we tried to improve mitochondrial pathway by increasing mitochondrial pyruvate pool via overexpression of the MPC complex^17^. However, the majority of pyruvate still existed in the cytoplasm and converted to ethanol. Therefore, transport of cytosolic pyruvate to the mitochondria is a limiting factor for efficient production of isobutanol via mitochondrial pathway.

In this study, we developed *S*. *cerevisiae* strain with enhanced isobutanol production using artificial cytosolic isobutanol biosynthetic pathway. In a previous study, a cytosolic isobutanol production pathway was constructed by overexpressing codon-optimized Ilv2, Ilv5, and Ilv3 in the cytoplasm while deleting the innate *ILV2* gene^8^. In this study, we found that *B*. *subtilis* (*Bs*) ALS (AlsS) is more active than the Ilv2 targeted to the cytosol, but overexpression of *Bs alsS*, and the subsequence accumulation of the pathway intermediate α-acetolactate led to a growth inhibition in *S*. *cerevisiae*. The growth inhibition could be alleviated when the downstream enzymes, Ilv5 and Ilv3, were expressed in the cytosol. To circumvent the growth inhibitory effect of α-acetolactate, we expressed *Bs alsS* under the control of copper-inducible *CUP1* promoter. Furthermore, we developed methods selecting efficient isobutanol-producing strains with multi-copy integration of the *Bs alsS* and cytosol-targeted *ILV5* and *ILV3* genes, based on their opposite effects on cell growth when overexpressed.

## Results and Discussion

### Construction of a cytosolic isobutanol biosynthetic pathway in *S*. *cerevisiae*

**Figure 2:**
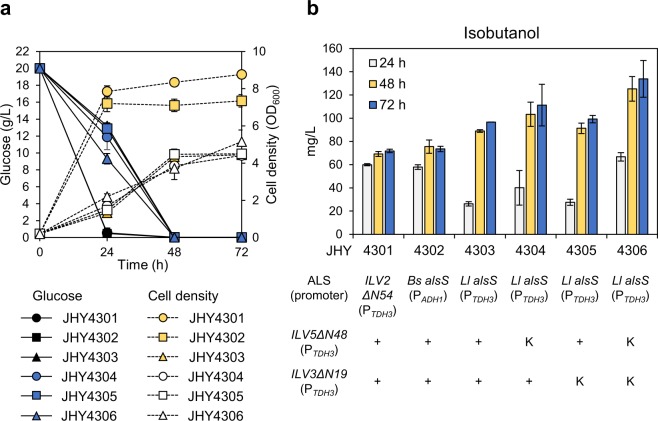
The effects of overexpressing various *ALS* genes and introducing Kozak sequence to *ILV5VN48* and *ILV3ΔN19*. (**a**) The indicated cells were grown in SC-His, Trp, Ura medium containing 2% glucose for 72 h, and cell growth and glucose uptake rate were monitored. (**b**) Isobutanol production levels were detected by HPLC. Each value indicates the average ± SD of triplicate experiments.

The innate isobutanol biosynthetic pathway in *S*. *cerevisiae* is subdivided into the cytosolic and mitochondrial pathways (Fig. 1). Therefore, to increase isobutanol production, sufficient amount of pyruvate should be introduced into the mitochondria, but pyruvate is mainly fermented to ethanol in the cytoplasm under most culture conditions^18^. To overcome this compartmentalization problem, we relocated llv2, Ilv5, and Ilv3 to the cytosol by deleting the N-terminal mitochondrial targeting sequence as reported previously^8^. *ILV2ΔN54*, *ILV5ΔN48*, and *ILV3ΔN19* were expressed under the control of a strong constitutive promoter, P_*TDH3*_, on CEN-based plasmids, in *bat1Δald6Δ* strain (JHY43), where the competing pathway genes, *BAT1* and *ALD6* involved in valine biosynthesis from 2-KIV and oxidation of isobutyraldehyde, respectively, were deleted^17^. JHY43 overexpressing *ILV2ΔN54*, *ILV5ΔN48*, and *ILV3ΔN19* (JHY4301) produced 71.8 mg/L isobutanol (Fig. 2b) after 72 h culture. We next compared the activity of Ilv2ΔN54 with other ALSs from *B*. *subtilis* and *Lactococcus lactis*. The *alsS* genes from *B*. *subtilis* (*Bs alsS*) and *L*. *lactis* (*Ll alsS*) were cloned into p413GPD plasmid vector providing P_*TDH3*_-driven gene expression, resulting in p413GPD-alsS(B) and p413GPD-alsS(L), respectively. Each plasmid was introduced into JHY43 together with the expression vectors for *ILV5ΔN48* and *ILV3ΔN19*. However, we could not obtain any transformants harboring p413GDP-alsS(B). Therefore, *Bs alsS* was expressed using a weaker promoter P_*ADH1*_ by cloning the gene into p413ADH vector. JHY43 cells harboring p413ADH-alsS(B) (JHY4302) showed similar isobutanol production level compared to that of JHY4301 (Fig. 2b). However, JHY43 harboring p413GPD-alsS(L) (JHY4303), showed an increase in isobutanol production up to 96.6 mg/L (Fig. 2b). JHY4303 showed a growth defect compared with JHY4301 and 4302, suggesting that higher ALS activity might inhibit cell growth (Fig. 2a). The accumulation of α-acetolactate is toxic to *S*. *cerevisiae* (Supplementary Fig. S2). Considering the more severe effect of *Bs alsS* on growth inhibition when expressed using the *TDH3* promoter_,_ *Bs* AlsS might have the highest enzyme activity followed by *Ll* AlsS and Ilv2ΔN54 in *S*. *cerevisiae*. We confirmed the different enzymatic activities of the three ALSs by introducing α-acetolactate decarboxylase (AlsD) from *B*. *subtilis*, which convert α-acetolactate to acetoin^19^. Acetoin can be further converted to 2,3-butanediol by endogenous enzyme Bdh1^20^. When the *ILV2ΔN54*, *Ll alsS*, or *Bs alsS* gene was expressed under the control of *TDH3* promoter together with *alsD*, JHY43 strain overexpressing *Bs alsS* produced the highest levels of acetoin and 2,3-butanediol followed by strains overexpressing *Ll alsS* and *ILV2ΔN54* (Supplementary Fig. S1). Ethanol production levels showed a negative correlation with the acetoin/2,3-butanediol levels, reflecting the competition between pyruvate decarboxylase (Pdc) and Als. (Supplementary Fig. S1).

### Increase in isobutanol production by introducing a Kozak sequence into ketol-acid reductoisomerase and dihydroxy-acid dehydratase

The ALS is the enzyme competing with pyruvate decarboxylase (PDC) for pyruvate availability (Fig. 1). Therefore, strong ALS activity might be the key to redirect the pyruvate flux from ethanol production to isobutanol production. *Bs alsS* was successfully used to produce acetoin in *S*. *cerevisiae* when the gene was overexpressed together with *B*. *subtilis alsD* encoding α-acetolactate decarboxylase^19^. However, the toxic effect of *Bs alsS* overexpression together with the overexpression of *ILV5ΔN48* and *ILV3ΔN19*, suggests that the following enzymes might not be active enough to convert the toxic intermediate generated by *Bs* AlsS. Since the transcription of *ILV5ΔN48* and *ILV3ΔN19* were already driven by the strong *TDH3* promoter, we tried to increase the protein expression levels by improving their translational efficiency. To do this, Kozak sequence was introduced into each gene. In eukaryotes including *S*. *cerevisiae*, 5′ untranslated region (5′-UTR) plays an important role in translational initiation. Especially the Kozak sequence rich in adenine is localized from the start codon to the −6 position of the 5′-UTR in *S*. *cerevisiae*^21,22^. Therefore, we introduced five adenine sequences in front of the start codon (AAAAAATG) to increase the protein expression levels of the *ILV5ΔN48* and *ILV3ΔN19* genes (Supplementary Fig. S3). Expression vectors for *ILV5ΔN48* and *ILV3ΔN19* containing the Kozak sequence, (K)*ILV5ΔN48* and (K)*ILV3ΔN19*, were introduced into JHY43 together with p413GPD-alsS(L). Compared with JHY4303 where *ILV5ΔN48* and *ILV3ΔN19* were expressed without Kozak sequence, JHY4306 expressing (K)*ILV5ΔN48* and (K)*ILV3ΔN19* produced about 1.4-fold higher level of isobutanol (133.9 mg/L), supporting the positive effect of increasing their protein expression levels in isobutanol production (Fig. 2b).

### Enhancing isobutanol production by overexpressing *alsS* from *B*. *subtilis* using copper-inducible promoter, P_*CUP1*_

**Figure 3:**
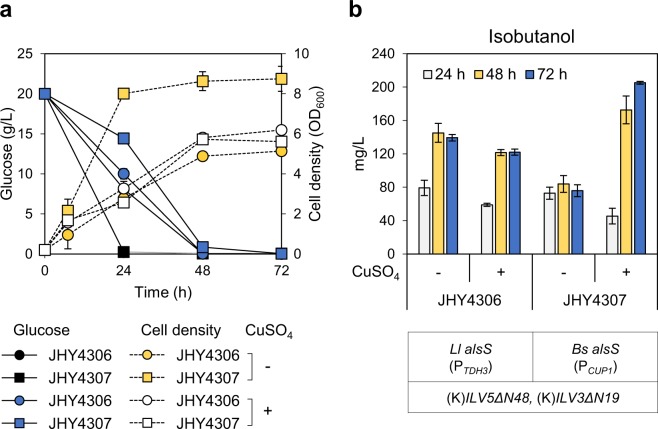
Expression of *alsS* from *B*. *subtilis* using copper-inducible promoter, P_*CUP1*_. JHY4306 and JHY4307 cells were grown in SC-His, Trp, Ura medium containing 2% glucose for 7 h and 100 μM of CuSO_4_ was added in the medium. Cells were further cultivated for 72 h, and the cell growth and glucose consumption (**a**) and Isobutanol production (**b**) were monitored. Each value indicates the average ± SD of duplicate experiments.

Although we could improve isobutanol production using *Ll* AlsS, the production level was still very low. Since *Bs* AlsS might have higher activity than *Ll* AlsS, but cannot be expressed using a strong constitutive promoter, we next considered using inducible promoter to overexpress *Bs alsS* at a specific time point. We chose the promoter of *CUP1* gene encoding metallothionein, which is activated by Ace1 transcription factor in the presence of copper ions^23–25^. To express *Bs alsS* using P_*CUP*1,_ P_*ADH1*_ in p413ADH-alsS(B) plasmid was replaced with P_*CUP1*_, and the resulting plasmid p413CUP1-alsS(B) was introduced into JHY43 strain together with the expression plasmids for (K)*ILV5ΔN48* and (K)*ILV3ΔN19*, generating JHY4307. JHY4306, where p413GPD-als(L) was introduced instead of p413CUP1-alsS(B), was used as a control. Cells were cultured for 7 h and then induced with 100 μM CuSO_4_. Copper induction in JHY4307 strain resulted in growth inhibition (Fig. 3a), but led to a 2.4-fold increase in isobutanol production from 84 mg/L to 205.2 mg/L (Fig. 3). In the control JHY4306 strain, the isobutanol production levels, which were not significantly affected by copper, were lower than those of copper-induced JHY4307 (Fig. 3). These results indicate that copper-inducible expression of *Bs alsS* using the P_*CUP1*_ is more effective in isobutanol production than constitutive expression of *Ll alsS* using the strong P_*TDH3*_ (Fig. 3).

### Multi-copy integration of *Bs alsS* at delta-sequences using a screening method based on the toxic effects of copper-dependent induction of *Bs alsS*

**Figure 4:**
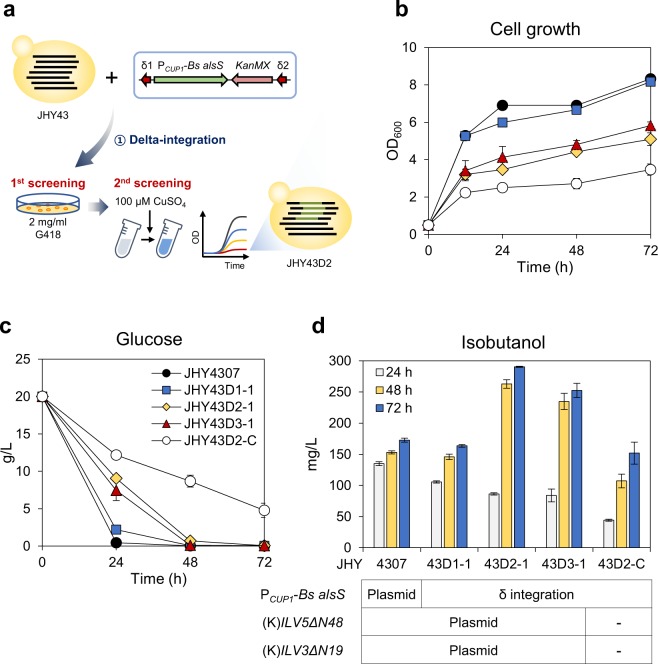
Multi-copy integration of *Bs alsS* into chromosome at delta-sequences (**a**) Experimental design to select yeast cells with multi-copy integration of *Bs alsS*. The delta-integration cassette containing *Bs alsS* and *KanMX* was introduced into JHY43, and selected in YPD medium containing 2 mg/ml G418. The selected transformants were treated with 100 μM of CuSO_4_, and the cells with reduced growth rate were selected. (**b**) The selected *Bs alsS*-integrated cells harboring plasmids p414GPD-(K)ILV5ΔN48, p416GPD-(K)ILV3ΔN19, and p413GPD (JHY43D1-1, JHY43D2-1, and JHY43D3-1), JHY43D2 harboring empty vectors (JHY43D2-C), and JHY4307 control expressing *Bs alsS* on p413CUP1-alsS(B) were inoculated to OD_600_ of 0.5 and were cultured in SC-His, Trp, Ura medium containing of 20 g/L glucose for 12 h, and 20 μM of CuSO_4_ was treated. Cells were further cultivated for 72 h. (**c**) Glucose consumption. (**d**) Isobutanol production. Each value indicates the average ± SD of triplicate experiments.

For more efficient production of isobutanol through regulating the expression levels of *Bs alsS*, we tried multi-copy integration of the P_*CUP1*_-controlled *Bs alsS* at delta-sequences, the retrotransposon Ty1 long terminal repeats (LTR), existing hundreds copies in the *S*. *cerevisiae* genome^26,27^. The delta-integration cassette containing P_*CUP1*_-*Bs alsS-*T_*CYC1*_ and G418-resistant marker (*KanMX*) was introduced into JHY43, and the transformants were first selected with high concentration of G418 (2 mg/ml), which might provide a selection power for the transformants with multiple *KanMX* marker genes (Fig. 4a). Next, a second screening of the strains with multiple integration of *Bs alsS* was performed based on the growth inhibitory effect of *Bs alsS* overexpression. The integrated copy number of the P_*CUP1*_-*Bs alsS* is expected to have a negative correlation with the cell growth rate upon inducing the expression of *Bs alsS* by copper. Therefore, the 26 transformants selected on G418 plate were cultured for 7 h and then 100 μM CuSO_4_ was added to the culture medium to induce the expression of *Bs alsS*. From this screening, JHY43D1, JHY43D2, and JHY43D3, showing significantly lower growth rates than that of JHY43 were selected (Supplementary Fig. S4).

Isobutanol production in the selected strains was investigated after introducing the expression plasmids for (K)*ILV5ΔN48* and (K)*ILV3ΔN19* into the strains JHY43D1, JHY43D2, and JHY3D3, resulting in JHY43D1-1, JHY43D2-1, and JHY43D3-1. Strain JHY4307 that expresses *Bs alsS* on p413CUP1-alsS(B) was used as a control. Cells were cultured for 12 h and the expression of *Bs alsS* was induced by adding 20 μM of CuSO_4_. JHY43D2-1 and JHY43D3-1 showed lower growth and glucose uptake rates compared with JHY4307 and JHY43D1-1 (Fig. 4b,c), but produced more isobutanol (Fig. 4d), reflecting a negative correlation between the cell growth and *Bs alsS* expression levels. JHY43D2-1 strain produced the highest level of isobutanol (290.4 mg/L), which is 1.7-fold higher than that produced in JHY4307 (172.7 mg/L), and even higher than that produced in JHY4307 after induction with 100 μM of CuSO_4_ (Fig. 3B). The best producer JHY43D2 contains about 4 copies of *Bs alsS* gene when the integrated gene copy number was determined by qPCR compared with JHY43DC, where one copy of *Bs alsS* gene was integrated into the *URA3* locus (Supplementary Fig. S6a). Taken together, the strains with multiple integration of *Bs alsS* at delta-sequences were successfully isolated based on the growth inhibitory effect *Bs alsS*.

### Assembly of the cytosolic isobutanol biosynthetic pathway by multi-copy integration of *ILV5ΔN48* and *ILV3ΔN19* genes at rDNA sites

**Figure 5:**
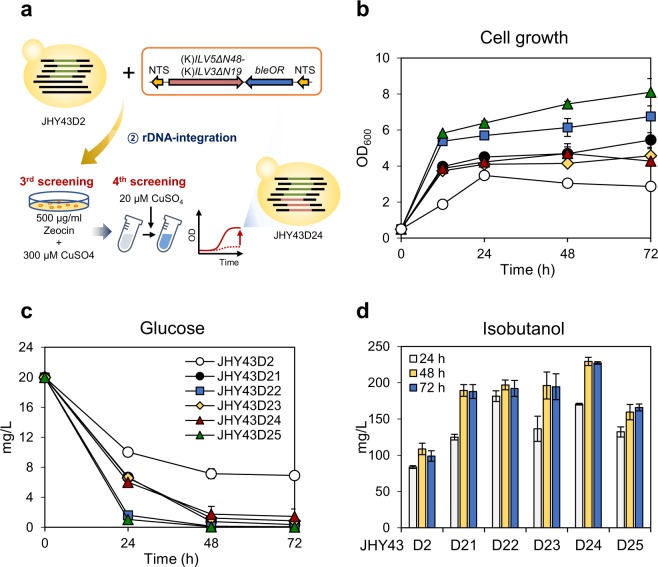
Integration of (K)*ILV5ΔN48* and (K)*ILV3ΔN19* at NTS sites. (**a**) Experimental design for multi-copy integration of (K)*ILV5ΔN48* and (K)*ILV3ΔN19* at NTS sites. NTS-integration cassette containing (K)*ILV5ΔN48*, (K)*ILV3ΔN19*, and *bleOR* marker was introduced into JHY43D2 strain and selected on YPD medium containing 500 μg/ml zeocin and 300 μg/ml CuSO_4_. The selected transformants were further screened for improved cell growth upon induction of *Bs alsS* by adding 20 μM of CuSO_4_. (**b**) The JHY43D2 control and the selected strains JHY43D21, JHY43D22, JHY43D23, JHY43D24 and JHY43D25 were inoculated to OD_600_ of 0.5 and cultured in SC mix medium containing 20 g/L glucose for 12 h, and treated with 20 μM CuSO_4_. Cells were further cultivated for 72 h. (**c**) Glucose consumption. (**d**) Isobutanol production. Each value indicates the average ± SD of triplicate experiments.

JHY43D2 strain harboring empty vectors (JHY43D2-C) showed a severe growth defect, which was relieved by overexpression of (K)*ILV5ΔN48* and (K)*ILV3ΔN19* (JHY43D2-1) (Fig. 4b). Overexpression of *ILV5ΔN48* and *ILV3ΔN19* might convert the toxic intermediate produced by *Bs* AlsS to less toxic isobutanol and other intermediates. We used this phenotype to generate stable isobutanol-production strain. To allow multiple integration of (K)*ILV5ΔN48* and (K)*ILV3ΔN19* genes into the JHY43D2 genome, rDNA (ribosomal DNA) repeats were used as the integration sites. The rDNA locus is composed of 150 tandem copies of a 9.1 kb repeat unit containing nontranscribed spacers (*NTS1* and *NTS2*)^28^. The rDNA integration cassette containing P_*TDH3*_-(K)*ILV5ΔN48*-T_*CYC1*_-P_*TDH3*_-(K)*ILV3ΔN19*-T_*CYC1*_ and bleomycin selection marker (*bleOR*), flanked by *NTS1* homology arms, was introduced into JHY43D2 (Fig. 5a). The transformants with multiple integration of (K)*ILV5ΔN48* and (K)*ILV3ΔN19* were selected on YPD plate containing 500 μg/mL of zeocin and 300 μM CuSO_4_ (Fig. 5a) (Supplementary Fig. S5). Copper-dependent induction of *Bs alsS* is toxic to the cells, thus cells with multiple integration of (K)*ILV5ΔN48* and (K)*ILV3ΔN19* could be selected on the medium containing high concentration of CuSO_4_ based on their faster growth. Five selected clones were grown in SC medium for 12 h and then treated with 20 μM CuSO_4_. All clones showed higher final cell densities (Fig. 5b) and higher glucose uptake levels (Fig. 5c) compared with the parental strain JHY43D2, reflecting the successful alleviation of the growth defect of JHY43D2. JHY43D24 strain showed the highest level of isobutanol production up to 227.2 mg/L. qPCR analysis revealed integration of 3 copies of (K)*ILV5ΔN48* and (K)*ILV3ΔN19* genes in the genome of JHY43D24 (Supplementary Fig. S6b). JHY43D24-53 strain having additional copy of (K)*ILV5ΔN48* and (K)*ILV3ΔN19* produced less isobutanol than JHY43D24, suggesting that our screening strategy was effective in isolating strains with optimal gene copy numbers (Supplementary Fig. S7). Furthermore, JHY43D24 strain showed consistent isobutanol production level after several subcultures, suggesting the stability of the integrated genes into the delta and NTS sites (Supplementary Fig. S8).

### Enhancing isobutanol production in JHY43D24 by optimizing induction conditions of *Bs alsS*

**Figure 6:**
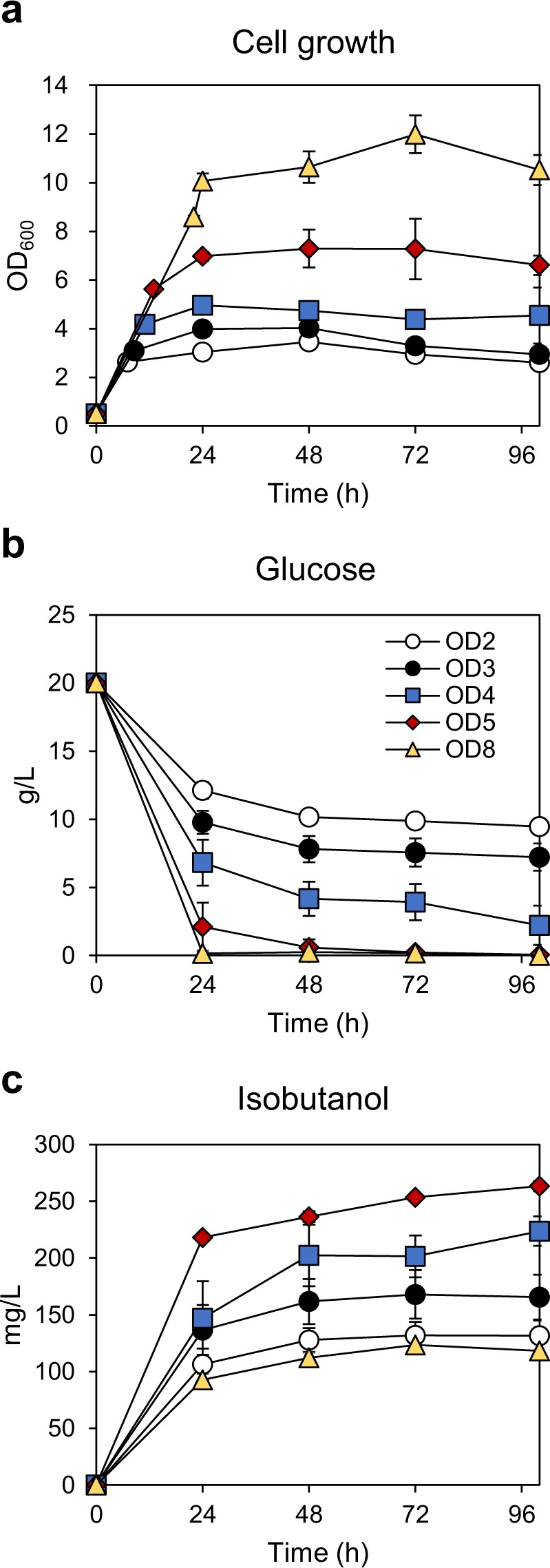
Optimization of copper induction time for isobutanol production. JHY43D24 cells were inoculated to OD_600_ of 0.5 and cultured in SC mix medium containing 20 g/L glucose. At the indicated cell densities, 20 μM of CuSO_4_ was added in the medium, and cell growth (**a**), glucose consumption (**b**), and isobutanol production (**c**) were monitored for 100 h. Each value indicates the average ± SD of triplicate experiments.

The expression of *Bs alsS* is critical for isobutanol production, but high level expression exerts a negative effect though inhibiting cell growth. Therefore, isobutanol production could be optimized by regulating the expression time and expression level of *Bs alsS*. First, we determined optimal cell density to induce *alsS* expression. The final engineered strain JHY43D24 was cultured and *Bs alsS* expression was induced with 20 μM CuSO_4_ at various cell densities (Fig. 6). The earlier the induction, the stronger was the inhibition of growth and glucose uptake (Fig. 6a,b). Induction of *alsS* at OD_600_ of 5 led to the highest isobutanol production level (263.2 mg/L) (Fig. 6c). Induction of *alsS* at a later point of OD_600_ of 8 resulted in the best cell growth, but the isobutanol production level was the lowest among the tested induction conditions (Fig. 6).

**Figure 7:**
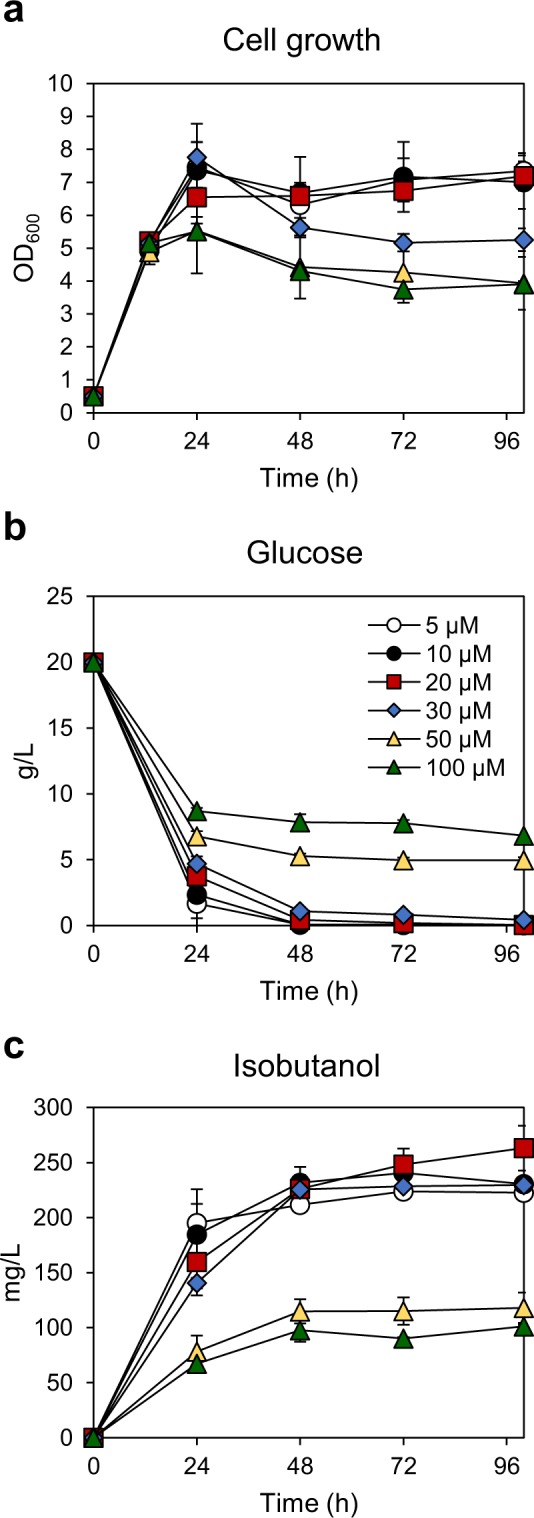
Optimization of CuSO_4_ concentration for isobutanol production. JHY43D24 cells were inoculated to OD_600_ of 0.5 and cultured in SC mix medium containing 20 g/L glucose. At OD_600_ of 5, various concentrations of CuSO_4_ were added in the medium, and cell growth (**a**), glucose consumption (**b**), and isobutanol production (**c**) were monitored for 100 h. Each value indicates the average ± SD of triplicate experiments.

Next, we tried to find the optimal concentration of CuSO_4_. JHY43D24 cells were grown to OD_600_ of 5 and *alsS* was induced with various concentrations of CuSO_4_ (Fig. 7). The cell growth and glucose consumption rates decreased as increasing the concentrations of CuSO_4_ (Fig. 7a,b). Although 5 μM CuSO_4_ was enough to induce *alsS* expression and subsequent isobutanol production, the highest titer of isobutanol (263.1 mg/L) was produced in cells induced with 20 μM CuSO_4_, which is the same concentration we used in Fig. 6 (Fig. 7c). In most culture conditions, ethanol was the prominent byproduct and acetoin accumulated over time (Supplementary Fig. S9).

## Conclusions

In this study, we engineered *S*. *cerevisiae* strain to produce isobutanol using an artificial cytosolic isobutanol biosynthetic pathway consisting of AlsS from *B*. *subtilis* and endogenous Ilv5 and Ilv3 targeted to the cytosol by deleting the mitochondrial targeting sequences. AlsS is a key enzyme converting the cytosolic pyruvate to α-acetolactate, competing with the ethanol production pathway. However, we found that excessive production of α-acetolactate by overexpressing of *Bs alsS* is toxic to the cells, which can be alleviated by subsequent conversion of α-acetolactate to other chemicals by Ilv5ΔN48 and Ilv3ΔN19. Therefore, to reduce growth inhibition mediated by accumulation of the toxic intermediate α-acetolactate, *Bs alsS* was expressed under the control of a copper-inducible *CUP1* promoter, and the translational efficiency of *ILV5ΔN48* and *ILV3ΔN19* was increased by adding Kozak sequence. Furthermore, the toxic effect of α-acetolactate was used to develop novel screening methods for multi-copy integration of the pathways genes. Multi-copy integration of *Bs alsS* into delta-sequences was screened based on the growth inhibition upon the induction of *Bs alsS*, whereas multi-copy integration of (K)*ILV5ΔN48* and (K)*ILV3ΔN19* with Kozak sequence into the rDNA sites of the *alsS*-integrated strain was screened based on the growth recovery effect. The strain JHY43D24 generated by this screening methods contains 4 copies of *Bs alsS* and 3 copies of (K)*ILV5ΔN48* and (K)*ILV3ΔN19*. After optimizing the *alsS* induction conditions, the final engineered strain produced 263.2 mg/L isobutanol, showing about 3.3-fold increase compared to the episomal expression of the cytosol-targeted endogenous genes *ILV2*, *ILV5*, and *ILV3* from strong constitutive promoters. However, the isobutanol production level is still very low compared to its production in other bacteria. Therefore, to further improve isobutanol production, it might be critical to improve the activities of Ilv5ΔN48 and Ilv3ΔN19, which require NADPH and iron-sulfur cluster, respectively.

## Methods

### Strains and culture conditions

All strains used in this study are described in Table 1. *E*. *coli* strain DH5α was used for construction of plasmids and cultured at 37 °C in Luria-Bertani (LB) medium containing 50 μg/mL of ampicillin. JHY43 strain, *BAT1* and *ALD6* deletion mutant derived from *S*. *cerevisiae* CEN. PK2-1C, was used as parental strain of all the engineered strains in this study^29^. Yeast cells were cultured in YPD medium or in synthetic complete (SC) medium (20 g/L glucose, 6.7 g/L yeast nitrogen base without amino acids, and 1.67 g/L amino acids mixture lacking histidine, tryptophan, leucine, and uracil) supplemented with auxotrophic nutrients as required.

**Table 1:** Strains used in this study.

Strain	Genotype	Reference
CEN. PK2-1C	*MATa ura3-52 trp1-289 leu2-3*,*112 his3 Δ1 MAL2-8* ^*C*^ *SUC2*	EUROSCARF
JHY43	CEN.PK2-1C *ald6Δ::loxP bat1Δ::loxP*	^29^
JHY4301	JHY43 harboring p413GPD-ILV2ΔN54, p414GPD-ILV5ΔN48, and p416GPD-ILV3ΔN19	This study
JHY4302	JHY43 harboring p413ADH-alsS(B), p414GPD-ILV5ΔN48, and p416GPD-ILV3ΔN19	This study
JHY4303	JHY43 harboring p413GPD-alsS(L), p414GPD-ILV5ΔN48, and p416GPD-ILV3ΔN19	This study
JHY4304	JHY43 harboring p413GPD-alsS(L), p414GPD-(K)ILV5ΔN48, and p416GPD-ILV3ΔN19	This study
JHY4305	JHY43 harboring p413GPD-alsS(L), p414GPD-ILV5ΔN48, and p416GPD-(K)ILV3ΔN19	This study
JHY4306	JHY43 harboring p413GPD-alsS(L), p414GPD-(K)ILV5ΔN48, and p416GPD-(K)ILV3ΔN19	This study
JHY4307	JHY43 harboring p413CUP1-alsS(B), p414GPD-(K)ILV5ΔN48, and p416GPD-(K)ILV3ΔN19	This study
JHY43DC	CEN.PK2-1C *ald6Δ::loxP bat1Δ::loxP URA3::* P_*CUP1*_*-Bs alsS-*T_*CYC1*_	This study
JHY43D1	JHY43 with random multiple integration of P_*CUP1*_*-Bs alsS-*T_*CYC1*_ at delta -sequences	This study
JHY43D2	JHY43 with random multiple integration of P_*CUP1*_*-Bs alsS-*T_*CYC1*_ at delta -sequences	This study
JHY43D3	JHY43 with random multiple integration of P_*CUP1*_*-Bs alsS-*T_*CYC1*_ at delta -sequences	This study
JHY43D1-1	JHY43D1 harboring p413GPD, p414GPD-(K)ILV5ΔN48, and p416GPD-(K)ILV3ΔN19	This study
JHY43D2-1	JHY43D2 harboring p413GPD, p414GPD-(K)ILV5ΔN48, and p416GPD-(K)ILV3ΔN19	This study
JHY43D2-C	JHY43D2 harboring p413GPD, p414GPD, and p416GPD	This study
JHY43D2-53	JHY43D2 with *HIS3::* P_*TDH3*_-(K)*ILV5ΔN48-*T_*CYC1*_, P_*TDH3*_*-*(K)*ILV3ΔN19-*T_*CYC1*_	This study
JHY43D3-1	JHY43D3 harboring p413GPD, p414GPD-(K)ILV5ΔN48, and p416GPD-(K)ILV3ΔN19	This study
JHY43D21	JHY43D2 with random multiple integration of P_*TDH3*_-(K)*ILV5ΔN48-*T_*CYC1*_, P_*TDH3*_*-*(K)*ILV3ΔN19-*T_*CYC1*_ at NTS sites	This study
JHY43D22	JHY43D2 with random multiple integration of P_*TDH3*_-(K)*ILV5ΔN48-*T_*CYC1*_, P_*TDH3*_*-*(K)*ILV3ΔN19-*T_*CYC1*_ at NTS sites	This study
JHY43D23	JHY43D2 with random multiple integration of P_*TDH3*_-(K)*ILV5ΔN48-*T_*CYC1*_, P_*TDH3*_*-*(K)*ILV3ΔN19-*T_*CYC1*_ at NTS sites	This study
JHY43D24	JHY43D2 with random multiple integration of P_*TDH3*_-(K)*ILV5ΔN48-*T_*CYC1*_, P_*TDH3*_*-*(K)*ILV3ΔN19-*T_*CYC1*_ at NTS sites	This study
JHY43D24-53	JHY43D24 with *HIS3::* P_*TDH3*_-(K)*ILV5ΔN48-*T_*CYC1*_, P_*TDH3*_*-*(K)*ILV3ΔN19-*T_*CYC1*_	This study
JHY43D25	JHY43D2 with random multiple integration of P_*TDH3*_-(K)*ILV5ΔN48-*T_*CYC1*_, P_*TDH3*_*-*(K)*ILV3ΔN19-*T_*CYC1*_ at NTS sites	This study

To produce istobutanol, yeast cells harboring proper plasmids were pre-cultured in 4 mL selective SC medium containing 20 g/L glucose, inoculated to OD_600_ of 0.2 or 0.5 in 6.5 mL of the same medium in 50 mL conical tube, and then cultured at 30 °C with constant shaking at 170 rpm. For copper induction, overnight culture cells were diluted to OD_600_ of 0.2 or 0.5, incubated for 7 h to 12 h in SC medium containing 20 g/L glucose, and then induced with appropriate concentrations of CuSO_4_.

### Construction of plasmids

Plasmids and primers used in this study are described in Table S1 and Table S2, respectively. The N-terminally truncated *ILV* genes (*ILV2*, *ILV5*, and *ILV3*) were obtained by PCR amplification from *S*. *cerevisiae* genomic DNA using specific primer pairs (ORF N F and ORF R), generating *ILV2ΔN54*, *ILV5ΔN48*, and *ILV3ΔN19*. These DNA fragments were cloned between *Bam*HI and *Xho*I sites of p413GPD, p414GPD, and p416GPD^30^, respectively, resulting in p413GPD-ILV2ΔN54, p414GPD-ILV5ΔN48, and p416GPD-ILV3ΔN19 plasmids. The ORF DNA fragments of *alsS*(B) from *B*. *subtilis*, and *alsS*(L) from *L*. *lactis* were amplified by PCR from each genomic DNA using target-specific primer pairs (ORF F and ORF R). To generate single-gene-expression plasmids, PCR-amplified products were cloned into p413GPD or p413ADH plasmids, resulting in p413GPD-alsS(L) and p413ADH-alsS(B). In p414GPD-ILV5ΔN48 and p416GPD-ILV3ΔN19 plasmid, *ILV5ΔN48* and *ILV3ΔN19* were replaced with the Kozak sequence*-ILV5ΔN48* and Kozak sequence-*ILV3ΔN19* fragments obtained by PCR amplification with primer pairs (ORF_K F and ORF R), generating p414GPD-(K)ILV5ΔN48 and p416GPD-(K)ILV3ΔN19 plasmids. Also, in p413ADH-alsS(B), P_*ADH1*_ part was replaced with P_*CUP1*_, generated by PCR with CUP1p F and CUP1p R primers, generating p413CUP1-alsS(B).

To construct a vector for delta-integration, Delta6M-alsS plasmid was constructed as previously described with minor modifications^31^. Two half fragments of YARCdelta4, YARCdelta4-1 (167 bp, fragment1) and YARCdelta4-2 (170 bp, fragment2), were obtained by PCR amplified from *S*. *cerevisiae* genomic DNA. *Amp*^*R*^-expression cassette (fragment3) was obtained by PCR from p413GPD. These 3 DNA fragments were combined by using overlapping PCR, and the resulting DNA fragment containing YARCdelta4-1, *Amp*^*R*^, and YARCdelta4-2 was cloned between the *Nhe*I and *Not*I sites of pUG6MCS^32^, resulting in Delta6M. Gene-expression cassette, P_*CUP1*_-*alsS (B)*-T_*CYC1*_, PCR-amplified from p413CUP1-alsS(B) was cloned between *Nhe*I and *Not*I sites of Delta6M, resulting in Delta6M-alsS.

To construct NTS66M-53 plasmid for NTS site-integration, two half DNA fragments of NTS1-2, NTS1-2a (400 bp) and NTS1-2b (400 bp), were amplified from *S*. *cerevisiae* genomic DNA. *Amp*^*R*^ cassette and *bleOR* cassette were obtained from pUG66 vector by PCR. These 4 PCR products were assembled by overlapping PCR, and the resulting DNA fragment, NTS1-2a-*bleOR-Amp*^*R*^-NTS1-2b, was ligated with *ILV5ΔN48*-expression cassette (P_*TDH3*_-Kozak sequence-*ILV5ΔN48*-T_*CYC1*_), resulting in NTS66M-5 plasmid. *ILV3ΔN19*-expression cassette (P_*TDH3*_-*Kozak sequence-ILV3ΔN19*-T_*CYC1*_) flanked by *Mau*BI and and *Not*I sites were cloned into *Asc*I and *Not*I site of the NTS66M-5, resulting in NTS66M-35 plasmid.

### Analytical methods

Cell growth was determined by the measurement of an optical density at 600 (OD_600_) with spectrophotometer (Varian Cary® 50 UV-Vis). To determine isobutanol, glucose, and ethanol concentrations, 500 μL of culture supernatants filtered through a 0.22 μm syringe filter were analyzed by High performance liquid chromatography (HPLC) following a previously described procedure^17^.

## Supplementary information

Supplementary materials
