# Publisher Correction: Development of an efficient cytosolic isobutanol production pathway in *Saccharomyces cerevisiae* by optimizing copy numbers and expression of the pathway genes based on the toxic effect of α-acetolactate

**DOI:** 10.1038/s41598-019-53786-y

**Published:** 2019-11-19

**Authors:** Seong-Hee Park, Ji-Sook Hahn

**Affiliations:** School of Chemical and Biological Engineering, Institute of Chemical Processes, Seoul National University, 1 Gwanak-ro, Gwanak-gu, Seoul, 08826 Republic of Korea

Correction to: *Scientific Reports* 10.1038/s41598-019-40631-5, published online 08 March 2019

In the original version of this Article, the incorrect Supplementary materials file was published. This has been corrected in the HTML version of the Article; the PDF version was correct at the time of publication.

