# Supplementary material for: Development of an efficient cytosolic isobutanol production pathway in Saccharomyces cerevisiae by optimizing copy numbers and expression of the pathway genes based on the toxic effect of α-acetolactate
Source: Sci Rep. 2019 Mar 8;9:3996. doi: 10.1038/s41598-019-40631-5 (PMC6408573; doi:10.1038/s41598-019-40631-5)
Supplement: Supplementary file 1 — Supplementary materials [file 41598_2019_40631_MOESM1_ESM.pdf]

Supplementary Materials

Development of an efficient cytosolic isobutanol production pathway in *Saccharomyces cerevisiae* by optimizing the copy numbers and expression of the pathway genes based on the toxic effect of α-acetolactate

Seong-Hee Park and Ji-Sook Hahn*

School of Chemical and Biological Engineering, Seoul National University, 1 Gwanak-ro, Gwanak-gu, Seoul 08826, Republic of Korea

*Corresponding author: Phone: +82-2-880-9228

Fax: +82-2-888-1604

e-mail: hahnjs@snu.ac.kr

Supplementary Table S1

Plasmids used in this study

Plasmid	Description	Reference
p413GPD	CEN/ARS plasmid, *HIS3*, $P_{TDH3,}$ T_{CYC1}	1
P414GPD	CEN/ARS plasmid, *TRP1*, $P_{TDH3,}$ T_{CYC1}	1
p416GPD	CEN/ARS plasmid, *URA3*, $P_{TDH3,}$ T_{CYC1}	1
p413GPD-ILV2ΔN54	CEN/ARS plasmid, *HIS3,* P_{TDH3}-*ILV2ΔN54*-T_{CYC1}	This study
p414GPD-ILV5ΔN48	CEN/ARS plasmid, *TRP1,* P_{TDH3}-*ILV5ΔN48*-T_{CYC1}	This study
p416GPD-ILV3ΔN19	CEN/ARS plasmid, *URA3,* P_{TDH3}-*ILV3ΔN19*-T_{CYC1}	This study
p413ADH-alsS(B)	CEN/ARS plasmid, *HIS3,* P_{ADH1}-*Bs alsS*-T_{CYC1}	This study
p413GPD-alsS(B)	CEN/ARS plasmid, *HIS3,* P_{TDH3}-*Bs alsS*-T_{CYC1}	This study
p413GPD-alsS(L)	CEN/ARS plasmid, *HIS3,* P_{TDH3}-*Ll alsS*-T_{CYC1}	This study
p414GPD-alsD(B)	CEN/ARS plasmid, *TRP1,* P_{TDH3}-*Bs alsD*-T_{CYC1}	This study
p414GPD-(K)ILV5ΔN48	CEN/ARS plasmid, *TRP1,* P_{TDH3}-(K)*ILV5ΔN48*-T_{CYC1}	This study
p416GPD-(K)ILV3ΔN19	CEN/ARS plasmid, *URA3,* P_{TDH3}- (K)*ILV3ΔN19*-T_{CYC1}	This study
p413CUP1-alsS(B)	CEN/ARS plasmid, *HIS3,* P_{CUP1}-*Bs alsS*-T_{CYC1}	This study
p413GPD-ILV5ΔN48-FLAG	CEN/ARS plasmid, *HIS3,* P_{TDH3}-*ILV5ΔN48*-FLAG-T_{CYC1}	This study
p413GPD-(K)ILV5ΔN48-FLAG	CEN/ARS plasmid, *HIS3,* P_{TDH3}-(K)*ILV5ΔN48*-FLAG-T_{CYC1}	This study
p413GPD-ILV3ΔN19-FLAG	CEN/ARS plasmid, *HIS3,* P_{TDH3}-*ILV3ΔN19*-FLAG-T_{CYC1}	This study
p413GPD-(K)ILV3ΔN19-FLAG	CEN/ARS plasmid, *HIS3,* P_{TDH3}-(K)*ILV3ΔN19*-FLAG-T_{CYC1}	This study
pUG6MCS	pUG6 plasmid containing additional restriction enzyme sites	2
Delta6M	Plasmid containing *loxP-KanMX-loxP* flanked by YARCdelta4-1 and YARCdelta4-2	This study
Delta6M-alsS	Delta6M plasmid, P_{CUP1}-*Bs alsS*-T_{CYC1}	This study
NTS66M-5	Plasmid containing *bleOR* flanked by NTS1-2a and NTS1-2b, P_{TDH3}-(K)*ILV5ΔN48*-T_{CYC1}	This study

NTS66M-53	Plasmid containing *bleOR* flanked by NTS1-2a and NTS1-2b, P_{TDH3}-(K)*ILV5ΔN48*-T_{CYC1}, P_{TDH3}-(K)*ILV3ΔN19*-T_{CYC1}	This study

Supplementary Table S2

Primers used for gene cloning

Primers	Sequ3ence (5’-3’)
ILV2ΔN54 F	AGCTGGATCCATGCCAGAGCCTGCTCCAAGTT
ILV2ΔN54 R	GCGCTCGAGTCAGTGCTTACCGCCTGTACGCT
ILV5ΔN48 F	CTGAGGATCCATGAAGCAAATCAACTTCGG
ILV5ΔN48 R	GATCCTCGAGTTATTGGTTTTCTGGTCTCAAC
ILV3ΔN19 F	GTACGGATCCATGGCAAAGAAGCTCAACAAGTA
ILV3ΔN19 R	TGCACTCGAGTCAAGCATCTAAAACACAACC
K_ILV5ΔN48 F	GCGGGATCCAAAAAATGAAGCAAATCAACTTCGG
K_ILV3ΔN19 F	GCGCTCGAGTTATGATTTATTTTGTTCAGCA
alsS (B) F	CTGAGGATCCATGACAAAAGCAACAAAAGAAC
alsS (B) R	CTGACTCGAGCTAGAGAGCTTTCGTTTTCA
alsS (L) F	GCGGGATCCATGGCACAATCCCTTCCC
alsS (L) R	GCGCTCGAGCTAATCTCCCCCCAATTCCA
alsD (B) F	CTGAGGATCCATGAAACGAGAAAGCAACAT
alsD (B) R	CTGACTCGAGTTATTCAGGGCTTCCTTCAG
ILV5ΔN48 FLAG R	GCGCTCGAGTTACTTGTCATCGTCGTCCTTGTAGTCTCCGGAACTACCTTGG TTTTCTGGTCTCAAC
ILV3ΔN19 FLAG R	GCGCTCGAGGCGTCACTTGTCATCGTCGTCCTTGTAGTCTCCGGAACTACC AGCATCTAAAACACAACCG
CUP1p F	GCGGAGCTCTAAGCCGATCCCATTACCGA
CUP1p R	GCGGGATCCTTTATGTGATGATTGATTGATTGAT
Delta1 R	ATAGCGGCCGCATGTTTATATTCATTGATCCTATTACA
Delta1 F	CACATTTCCCCGAAAAGTGCATTTAAATTGTTGGAATAGAAATCAACTATC
Amp-Ori F	GCACTTTTCGGGGAAATGTG
Amp-Ori R	CTCAACATTCACCCATTTCTCAATTTAAATCGCAGGAAAGAACATGTGAG
Delta2 R	TGAGAAATGGGTGAATGTTGAG
Delta2 F	GCGGCTAGCATAAAACGGAATGAGGAATAATC
Promoter up F	GCGGCTAGCGAGCTCGGAAACAGCTATGACCATGA
NTS term R	GCGGGCGCGCCGCGGCCGCTAAGGGTTCTCGAGAGCTC
TEF prom F	GCCAAAAATTTACTTCGCGCGGCCCGACATGGAGGCCCAGAAT
NTS1-2b F	GCGAAGTAAATTTTTGGCG
NTS1-2b Amp R	TTTCCCCGAAAAGTGCATTTAAATCTAGTTTCTTGGCTTCCTATG
NTS1-2a F	CCGAGCGTGAAAGGATTTGCC

NTS1-2a R	GCGGCTAGCCAACCATTCCATATCTGTTAAG
Univ F2	GACTCGCGCGCGGGAACAAAAGCTGGAGCTC
Univ R	GACTACGCGTGCGGCCGCTAATGGCGCGCCATAGGGCGAATTGGGTACC

Underline indicates restriction enzymatic sites

Supplementary Table S3

Primers used for qPCR

Primers	Sequence (5’-3’)
alsS (B) qPCR F	CGCACCCTCTTGAAATCGTT
alsS (B) qPCR R	CCGAGTGTTTGCATACCGTT
ILV5ΔN48 qPCR F	CCCTCAGAATGGCAGAGGAA
ILV5ΔN48 qPCR R	GACGCACTGTTTCACCATCA
ILV3ΔN19 qPCR F	CTAGCTGCACCTTTGGAACC
ILV3ΔN19 qPCR R	GAGCGAATCCCAGTCAATCG
ACT1 qPCR F	GCCGAAAGAATGCAAAAGGA
ACT1 qPCR R	TAGAACCACCAATCCAGACGG

Supplementary method

Western blot analysis

Cells were broken with glass beads (Sigma-Aldrich. St. Louis, Missouri, USA) in 150 μL of IP150 buffer [50 mM Tris·HCl (pH 7.5), 150 mM NaCl, 2 mM MgCl2, 0.1% Nonidet P-40, and 0.1% protease inhibitor cocktail (EMD Millipor Calbiochem. MA, USA)]. Then proteins were boiled with adding 6 μL of 5 x sample loading dye [60 mM Tris·HCl (pH 6.8), 2 % SDS, 25 % (v/v) glycerol, 5 % β-mercaptoethanol, 0.1 % bromophenol blue] for 10 min, and separated by 8 % SDS-PAGE. The Ilv5ΔN48, (K)Ilv5ΔN48, Ilv3ΔN19, and (K)Ilv3ΔN19 proteins were analyzed by western blotting with an anti-DDDK-tag mAb (MBL Life Science) and anti-mouse IgG (Sigma-Aldrich. St. Louis, Missouri, USA). Hxk protein was detected by immunoblotting with anti-hexokinase (USBiological Life Sciences) and anti-rabbit IgG (Therm Fisher Scientific. Massachusetts, USA).

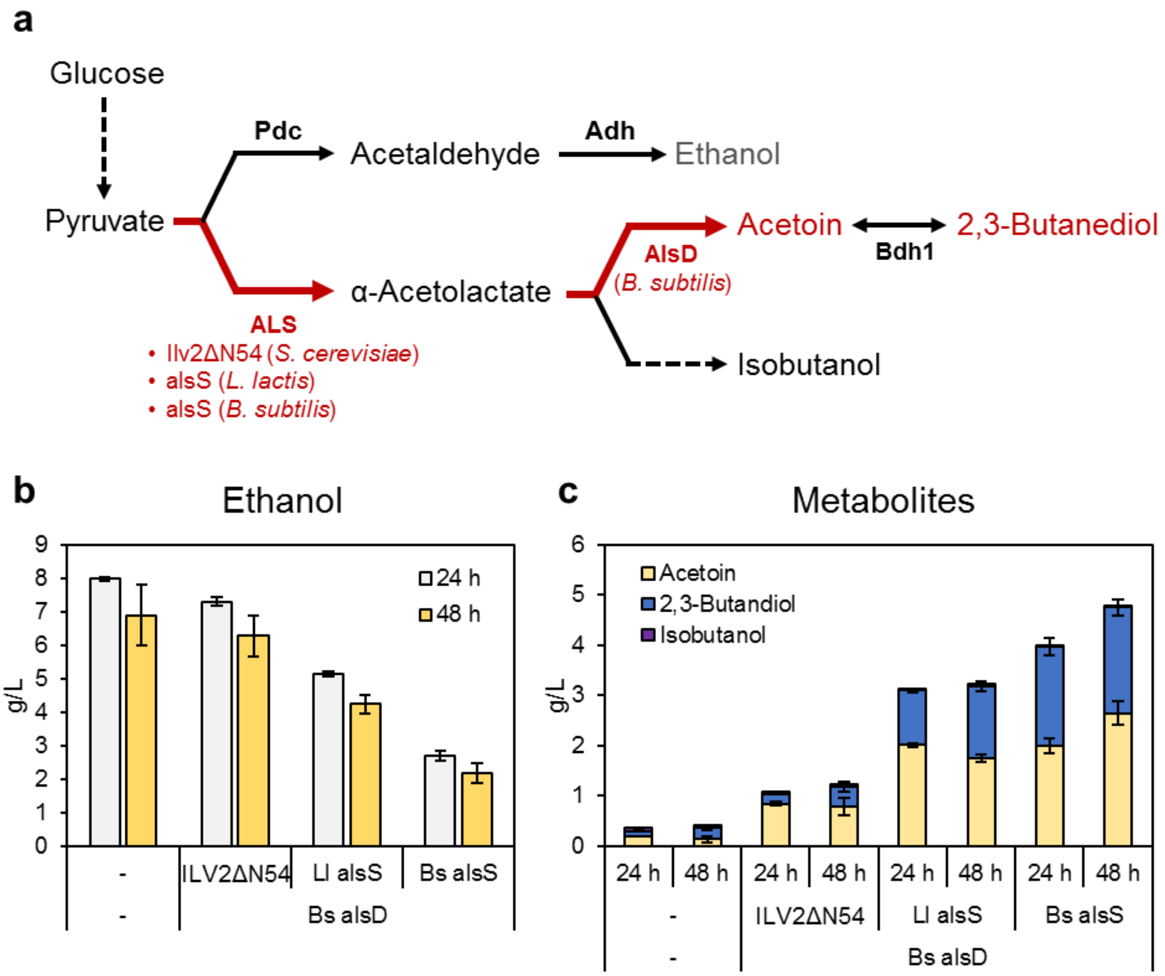

Supplementary Figure S1. Comparing activity of ALS from *S. cerevisiae*, *L. lactis*, and *B. subtilis* by detecting acetoin and 2,3-butanediol production. (a) Biosynthesis of pyruvate derivatives, isobutanol, ethanol, acetoin, and 2,3-butanediol in *S. cerevisiae*. The p413GPD-ILV2ΔN54, p413GPD-alsS(L), or p413GPD-alsS(B) plasmids were transformed in JHY43 cells with p414GPD-alsD(B). Cells were cultured in SC- His, Trp medium containing 2 % glucose, and ethanol (b), isobutanol, acetoin, and 2,3- butanediol (c) levels were monitored for 48 h. Each value indicates the average ± SD of duplicate experiments.

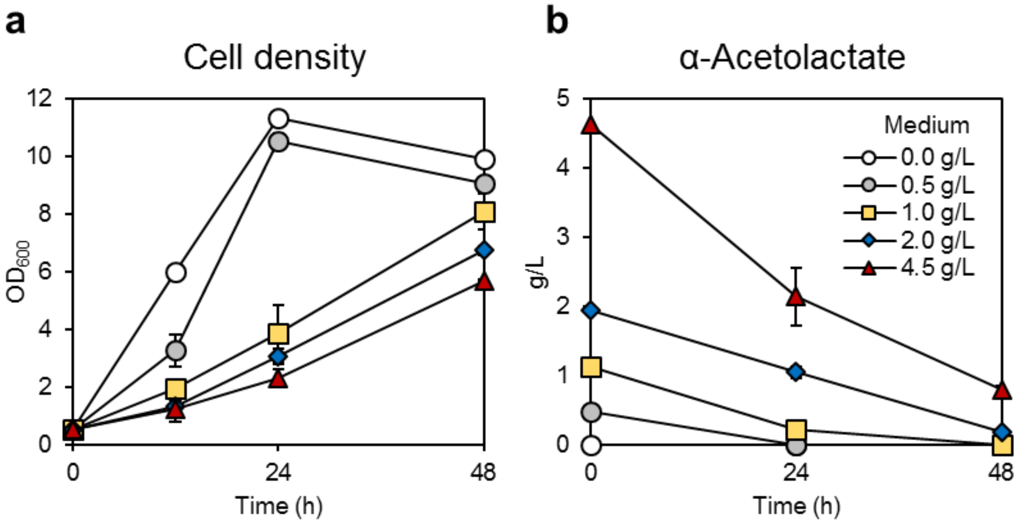

Supplementary Figure S2. α-Acetolactate toxicity to *S. cerevisiae* strain. JHY43 cells were inoculated to OD_{600} of 0.5 in 1 mL SC medium containing 2 % glucose and different concentrations of α-acetolactate. Cell growth (a) and α-acetolactate consumption (b) were monitored for 48 h. Each value indicates the average ± SD of duplicate experiments. DL-acetolactic acid (Santa Cruz Biotechnology, Inc. Texas, USA) was used as a standard and medium additive.

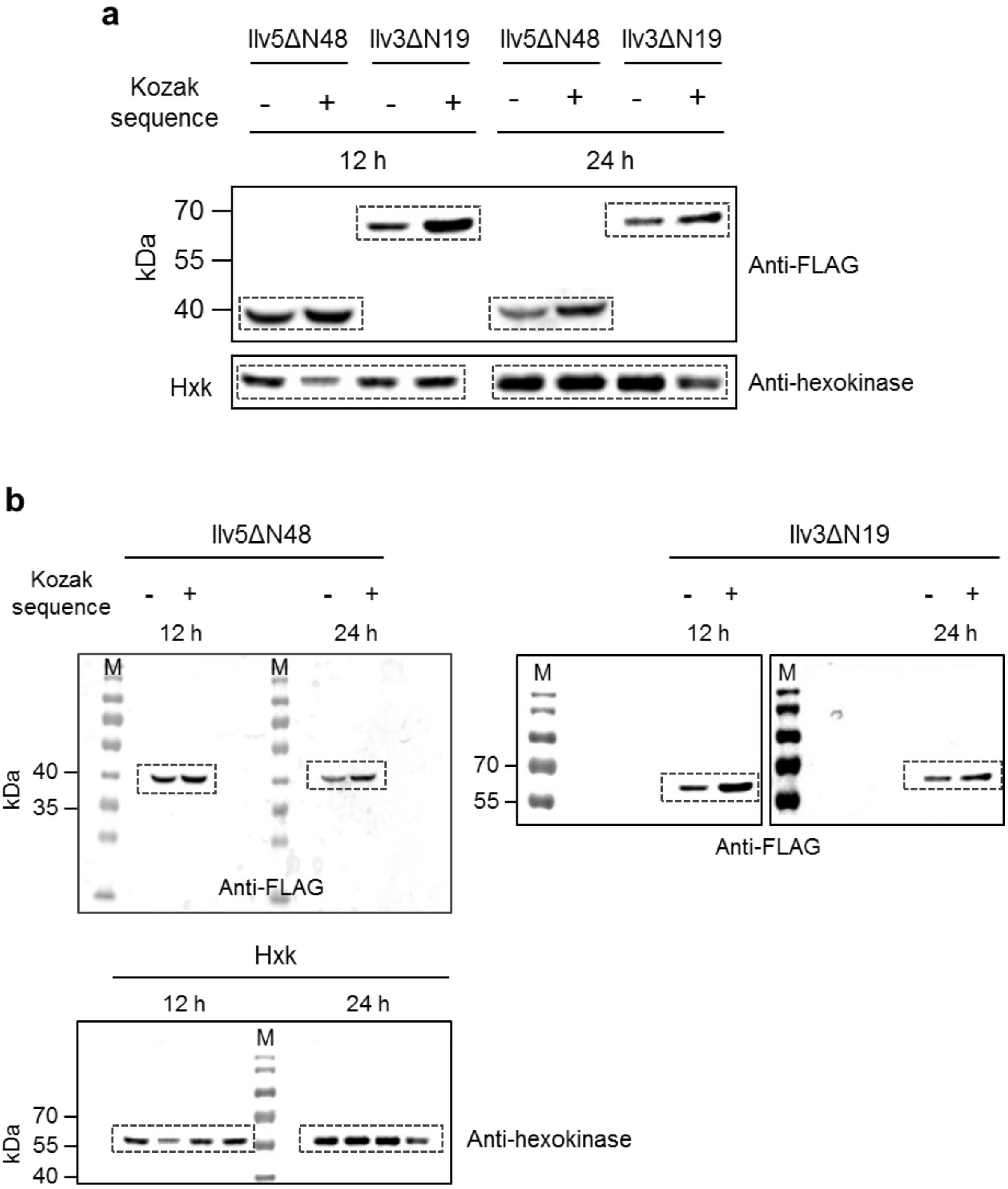

Supplementary Figure S3. Expression levels of Ilv5ΔN48 and Ilv3ΔN19 proteins with or without Kozak sequence. (a) JHY43 cells harboring p413GPD-ILV5ΔN48-FLAG, p413GPD-(K)ILV5ΔN48-FLAG, p413GPD-ILV3ΔN19-FLAG, or p413GPD-(K)ILV3ΔN19-FLAG were grown in SC-His containing 2 % glucose for 24 h, and Ilv5ΔN48-FLAG and Ilv3ΔN19-FLAG protein levels were detected by western blotting with anti-Flag antibody. Hxk protein was detected as a control. (b) Uncropped full-length blots presented in the Supplementary Figure S3a. Capital letter M represents loading of prestained protein ladder. Cropped areas in Figures boxed in dashed lines.

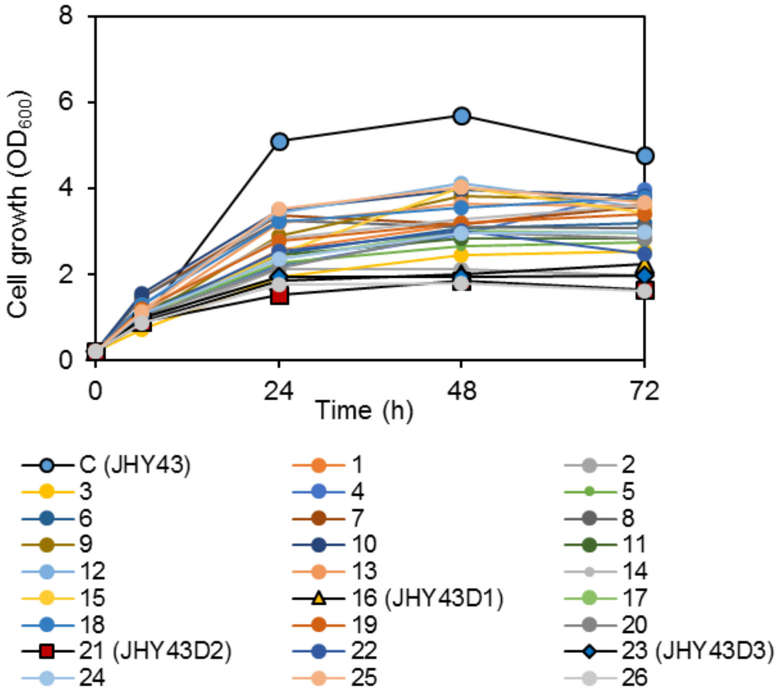

Supplementary Figure S4. Screening of JHY43 strains with multi-copy integration of *alsS* from *B. subtilis* at delta-sequences. The delta-integration cassette containing *Bs alsS* and *KanMX* was introduced into JHY43, and selected in YPD medium containing 2 mg/ml G418. The selected transformants were inoculated to OD_{600} of 0.2 and were cultured in SC mix medium containing of 20 g/L glucose. After 7 h, $CuSO_4$ was treated at a concentration of 100 µM, and strains with reduced growth were selected.

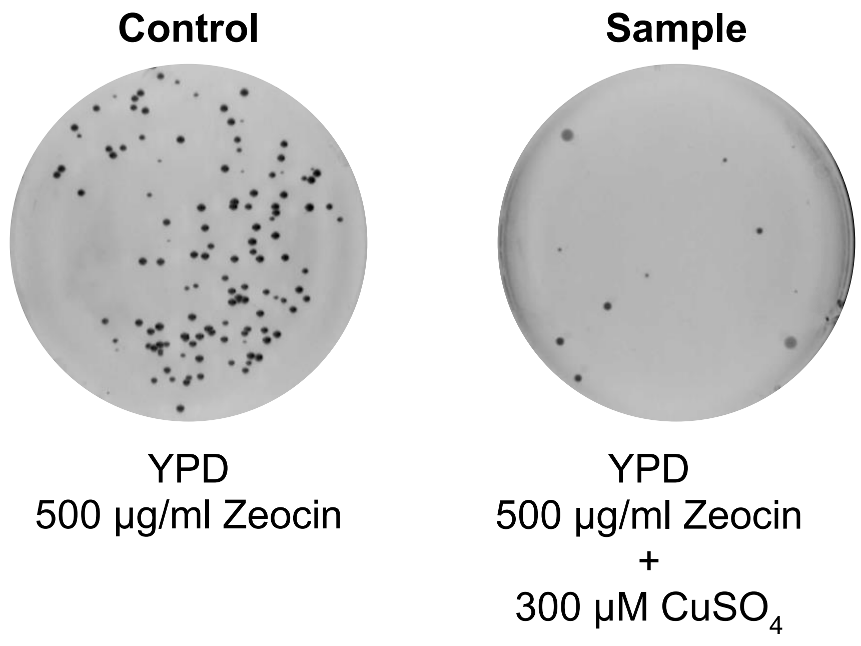

Supplementary Figure S5. Multi-copy integration of (K)*ILV5ΔN48* and (K)*ILV3ΔN19* genes at rDNA sites. NTS-integration cassette containing (K)*ILV5ΔN48*, (K)*ILV3ΔN19*, and *bleOR* marker was introduced into JHY43D2 strain and selected on YPD medium containing 500 µg/ml zeocin. By addition of $CuSO_4$ in YPD plate, strains with multiple integration of (K)*ILV5ΔN48* and (K)*ILV3ΔN19* genes were selected based on their growth under the conditions of copper-dependent induction of *alsS*.

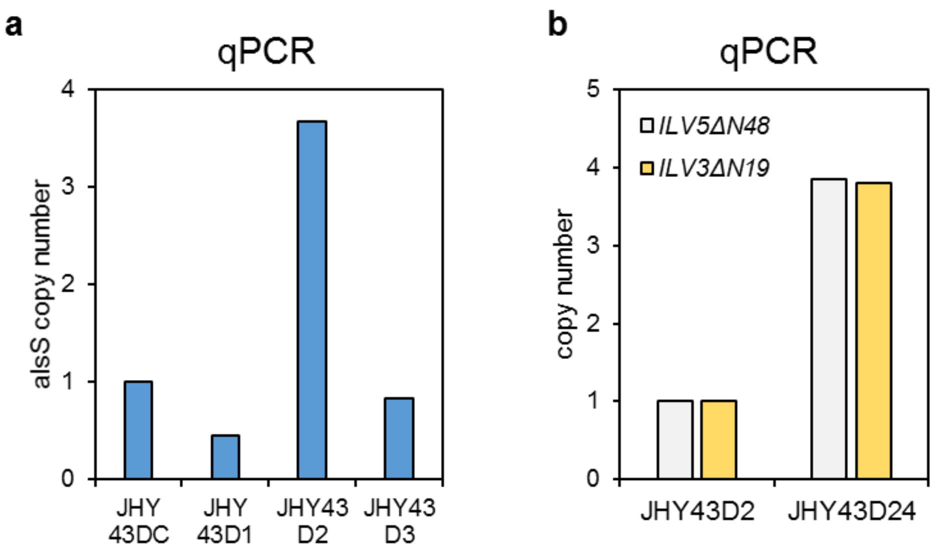

Supplementary Figure S6. Quantitative PCR (qPCR) to determine copy numbers of the integrated genes. The strains JHY43DC, JHY43D1, JHY43D2, JHY43D3, and JHY43D24 were cultured in YPD medium and genomic DNA was isolated. To determine the copy numbers of the integrated alsS gene in JHY43DC, JHY43D1, JHY43D2, JHY43D3, and the copy numbers of the integrated ILV5ΔN48 and ILV3ΔN19 genes in JHY43D24, qPCR analyses were performed. Reaction mixture containing 5 µl genomic DNA, 5 pmol each of gene-specific primers, and 1xSYBR Green I master mix (Roche Applied Science) was incubated with 45 cycles of 95°C for 40 s, 55°C for 20 s, and 72°C for 20 s on a LightCycler 480 II System (Roche Applied Science). The ACT1 housekeeping gene was used as a reference control. The crossing point (Cp) values were processed using LightCycler Software version 1.5 (Roche Applied Science) and expression levels were normalized as target/reference ratios. The qPCR primers used in this study are shown in Table S2. (A) The gene copy number of *alsS* from *B. subtilis* in JHY43 strain derivatives. JHY43DC strain is a control strain with one copy of *Bs alsS* integrated into *HIS3* site. (B) The gene copy number of (K)*ILV5ΔN48* and (K)*ILV3ΔN19* in JHY43D2.The gene copy number of JHY43D24 includes the endogenous *ILV5* and *ILV3*.

a

Strain	Copy number of *(K)ILV5ΔN48* and *(K)ILV3ΔN19*
JHY43D2	-
JHY43D2-53	1 copy
JHY43D24	3 copies
JHY43D24-53	4 copies

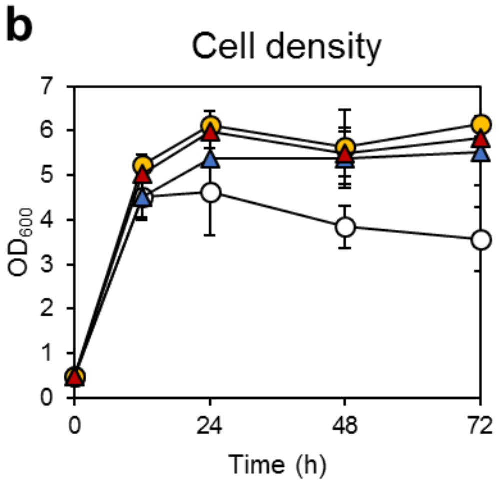

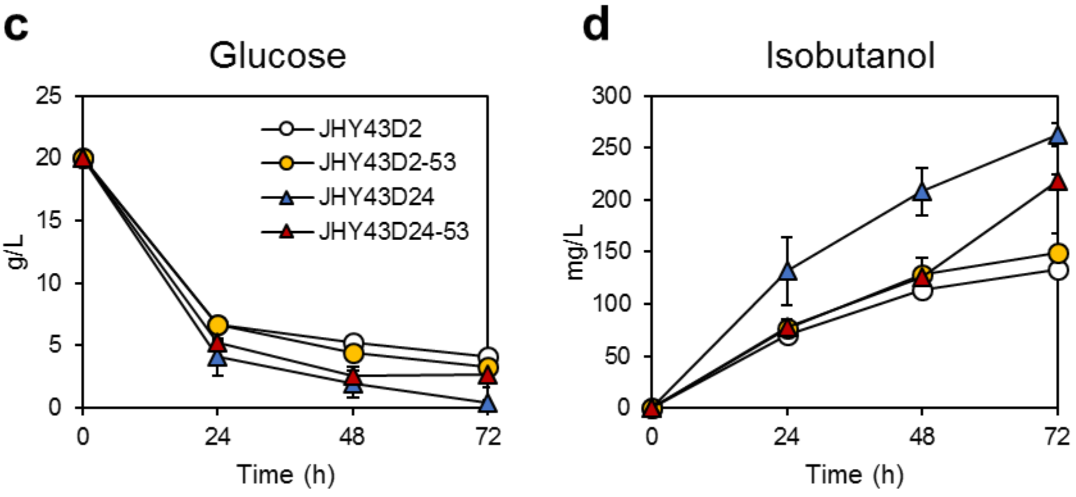

Supplementary Figure S7. Comparison of isobutanol production according to the number of copies of (K)*ILV5ΔN48* and (K)*ILV3ΔN19*. The JHY43D2-53 and JHY43D24-53 strains were constructed by inserting P_{TDH3}-(K)*ILV5ΔN48*-T_{CYC1}-P_{TDH3}-(K)*ILV3ΔN19*-T_{CYC1} fragment into *HIS3* locus in JHY43D2 or JHY43D24 strains, respectively. (a) The gene copy numbers of (K)*ILV5ΔN48*A and (K)*ILV3Δ*N19 in each strain are indicated. The JHY43D2, JHY43D2-53, JHY43D24, and JHY43D24-53 were inoculated to OD_{600} of 0.5 and cultured in SC mix medium containing 20 g/L glucose for 12 h, and then treated with 20 µM $CuSO_4$. Cell density (b), glucose consumption (c), and isobutanol production (d) were monitored for 72 h. Each value indicates the average ± SD of triplicate experiments.

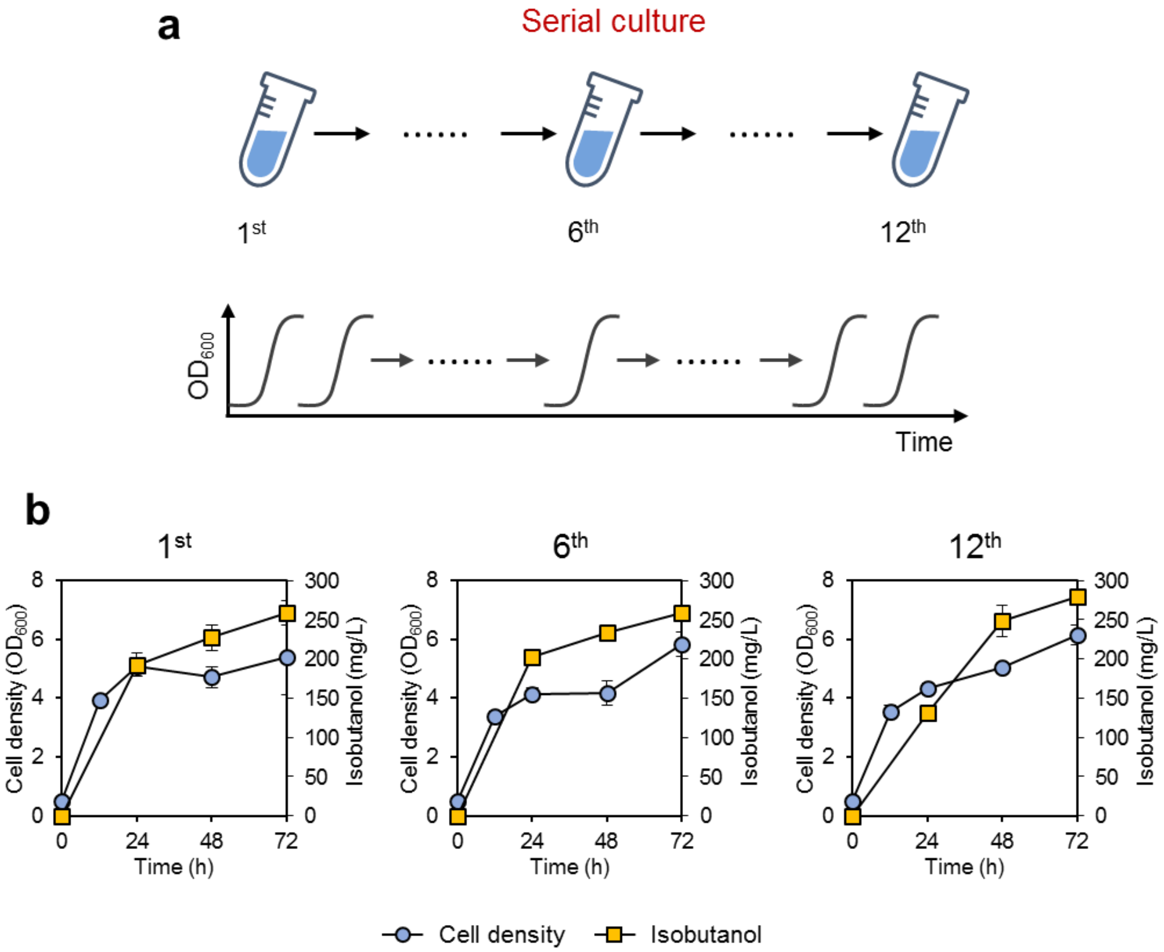

Supplementary Figure S8. Stability of the inserted genes in JHY43D24 strain. (a) A schematic diagram of the experiment. (b) JHY43D24 cells were subcultured in SC medium containing 2 % glucose for 12 times. The production of isobutanol in the first, sixth and twelfth subculture was detected. Twenty hours after the subculture, 20 µM $CuSO_4$ was treated. Each value indicates the average ± SD of duplicate experiments.

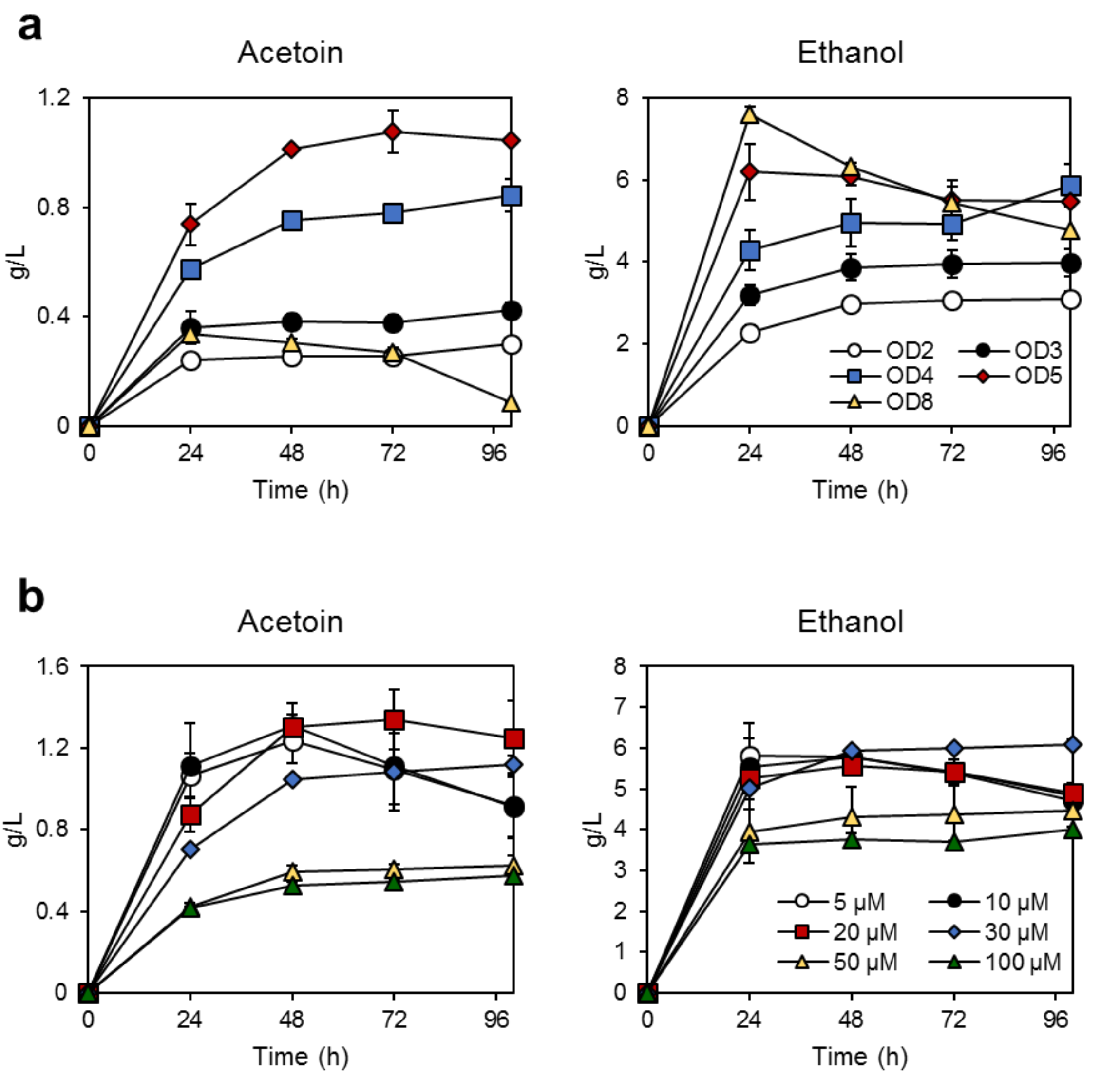

Supplementary Figure S9. Metabolites profiles of JHY43D24. (a) JHY43D24 cells were inoculated to OD_{600} of 0.5 and cultured in SC mix medium containing 20 g/L glucose. At the indicated cell densities, 20 μM of $CuSO_4$ was added in the medium. (b) JHY43D24 cells were inoculated to OD_{600} of 0.5 and cultured in SC mix medium containing 20 g/L glucose. At OD_{600} of 5, various concentrations of $CuSO_4$ were added in the medium. Acetoin and Ethanol production were monitored for 100 h. Each value indicates the average ± SD of triplicate experiments.

Supplementary references

1 Mumberg, D., Muller, R. & Funk, M. Yeast vectors for the controlled expression of heterologous proteins in different genetic backgrounds. *Gene* **156**, 119-122 (1995).

2 Baek, S. H. *et al.* Improvement of D-lactic acid production in *Saccharomyces cerevisiae* under acidic conditions by evolutionary and rational metabolic engineering. *Biotechnol J* **12** (2017).
